# Advances in Current Treatment Paradigms for Metastatic Hormone-Sensitive Prostate Cancer

**DOI:** 10.3390/jcm14082565

**Published:** 2025-04-08

**Authors:** Shayan Smani, Julien DuBois, Ismail Ajjawi, Nishan Sohoni, Ankur U. Choksi, Soum D. Lokeshwar, Isaac Y. Kim, Joseph F. Renzulli

**Affiliations:** Department of Urology, Yale School of Medicine, New Haven, CT 06520, USA; shayan.smani@yale.edu (S.S.); julien.dubois@yale.edu (J.D.); ismail.ajjawi@yale.edu (I.A.); nishan.sohoni@yale.edu (N.S.); ankur.choksi@yale.edu (A.U.C.); soum.lokeshwar@yale.edu (S.D.L.); isaac.kim@yale.edu (I.Y.K.)

**Keywords:** prostatic neoplasm, androgen deprivation therapy, metastasis, androgen receptor antagonists

## Abstract

Metastatic hormone-sensitive prostate cancer (mHSPCa) presents de novo or represents significant disease progression and requires systemic treatment. However, progression to castration resistance is inevitable. The treatment landscape has evolved with the introduction of intensified systemic therapy, including androgen deprivation therapy (ADT) combined with either androgen receptor signaling inhibitors (ARSIs) or cytotoxic chemotherapy (doublet therapy) or combined therapy with both agents (triplet therapy). Landmark trials such as CHAARTED, STAMPEDE, LATITUDE, ENZAMET, and TITAN have established combination therapies as the standard of care, demonstrating significant overall survival benefits. More recently, triplet therapy—integrating ADT, docetaxel, and an ARSI—has emerged as an effective approach, particularly in high-volume metastatic disease, as supported by ARASENS and PEACE-1. Advances in imaging, such as PSMA PET-CT, have improved disease detection, allowing earlier detection of metastasis and appropriate therapy. Similarly, genomic profiling has enabled biomarker-driven, personalized treatment strategies. The role of treatment of the primary tumor, by either radiation therapy or cytoreductive prostatectomy, in low-volume disease continues to be explored. As novel therapies, targeted agents, and immunotherapies undergo investigation, optimizing treatment selection based on disease burden, molecular characteristics, and patient factors will be essential. The future of mHSPCa management lies in multidisciplinary, precision-based approaches to improve patient outcomes while balancing treatment efficacy and tolerability.

## 1. Introduction

Prostate cancer (PCa) is a major global and national health concern. The American Cancer Society projects that in 2025, there will be approximately 314,000 new cases and 35,000 deaths due to PCa, making it the most commonly diagnosed, non-skin cancer in American men and the second leading cause of cancer-related mortality [1,2]. In recent years, the incidence of both localized and metastatic prostate cancer (PCa) has increased [2,3,4,5]. Notably, the annual rise in metastatic PCa has accelerated from 0.58% (2008–2012) to 2.74% (2012–2014), reaching 4–6% between 2015 and 2019 [6,7]. While the exact drivers of this trend remain uncertain, it may, in part, reflect the residual impact of the 2008 and 2012 U.S. Preventive Services Task Force (USPSTF) recommendations against routine PSA screening in asymptomatic men, resulting in more men presenting with advanced-stage disease [4,8,9].

While five-year disease-specific survival rates of localized PCa approach 99% [1,2,10], once PCa metastasizes, the prognosis worsens significantly, with a five-year disease-specific survival rate of just 37%, based on National Cancer Institute data from 2014–2020 [10]. Given ongoing advancements in metastatic prostate cancer treatment, a deeper understanding of the epidemiology, disease progression, and therapeutic strategies for metastatic prostate cancer and its various subtypes is crucial to improving patient outcomes.

Metastatic prostate cancer that no longer responds to conventional androgen deprivation therapy is classified as castration-resistant prostate cancer (CRPC). Rather than representing a formal stage progression, CRPC is best viewed as a recurrent disease characterized by disease progression despite castrate-level serum testosterone (traditionally <50 ng/dL) [11], often indicated by persistently elevated or rising PSA levels. CRPC carries a poorer prognosis than metastatic hormone sensitive prostate cancer (mHSPCa), with a median survival of 25.6 months after progression [12]. Currently, the preference would be to drive testosterone levels below 20 ng/dL, as recommended by the Bethesda Consensus group [13,14]. Consequently, preventing the development of castration-resistant disease and effectively treating those with mHSPCa is critical for improving overall outcomes.

Established non-modifiable risk factors for the development and severity of PCa include advanced age, Black race, family history, and the presence of germline mutations [15,16,17,18]. Germline mutations such as BRCA1/2, ATM, PTEN, and HOXB13, along with the cumulative impact of various single nucleotide polymorphisms (SNPs), have been identified as significant contributors and may shape future therapies targeting vulnerabilities in mHSPCa. As survival duration for metastatic PCa continues to improve, there is a growing focus on developing predictive models to identify patients at increased risk of rapid disease progression [19,20]. This aligns with the 2024 update to the National Comprehensive Cancer Network (NCCN) guidelines for prostate cancer and the 2023 revision of the American Urological Association (AUA) guidelines for advanced prostate cancer, both of which emphasize the adoption of novel targeted therapies and the integration of advanced imaging modalities to enhance disease characterization and guide individualized treatment selection [21,22]. This review aims to define mHSPCa and provide a comprehensive overview of landmark clinical trials to support clinicians in making evidence- based, patient-centered management decisions.

## 2. Classification and Diagnosis of mHSPCa

According to the AUA guidelines, mHSPCa is defined as PCa that has metastasized to distant sites but remains responsive to androgen deprivation therapy (ADT) [23]. Similarly, the National Comprehensive Cancer Network (NCCN) guidelines characterize mHSPCa as metastatic disease identified through imaging and clinical evaluation, typically managed using systemic therapies such as ADT in combination with additional treatment modalities [24].

The diagnosis of mHSPCa heavily relies on advanced imaging technologies for the accurate detection of metastatic lesions. While conventional imaging modalities such as computed tomography (CT) and bone scans are widely utilized as initial diagnostic tools, the broad adoption of advanced imaging techniques—particularly prostate-specific membrane antigen positron emission tomography (PSMA PET) and whole-body MRI—has markedly improved the sensitivity and specificity of detecting metastatic disease [25]. The landmark proPSMA trial, which included 203 men with high-risk localized prostate cancer, randomized participants to undergo either conventional imaging or PSMA PET-CT. Results demonstrated that PSMA PET-CT had significantly higher diagnostic accuracy (92%) compared to conventional imaging (65%) in identifying pelvic nodal or distant metastases [26]. Additionally, PSMA PET-CT reduced equivocal findings, led to more frequent management changes (28% vs. 15%), and resulted in lower radiation exposure. Subsequent studies have affirmed PSMA PET-CT as the gold standard for detecting both low-volume and high-volume disease due to its exceptional ability to identify micrometastatic disease that may be missed by conventional imaging [27,28,29]. Several alternative radioligands to PSMA have been investigated, including C-11/F-18 choline, F-18 piflufolastat, F-18 fluciclovine, and F-18 sodium fluoride, though they are not widely used in clinical practice [30]. Among these, C-11/F-18 choline is the most extensively studied; however, its relatively low sensitivity and positive predictive value limit its clinical utility [31].

A critical aspect of mHSPCa management is its classification into low-volume and high-volume disease, as this stratification significantly influences prognosis and treatment planning. This classification system, originally established by the CHAARTED trial, defines high-volume disease as the presence of visceral metastases or ≥4 bone metastases with ≥1 outside the vertebral column or pelvis [32]. Conversely, low-volume disease lacks these features. Patients with low-volume disease generally have a more favorable prognosis and may benefit from treatment approaches such as systemic therapy combined with localized interventions like radiotherapy or prostate surgery [33,34]. Patients with high-volume disease often face a more aggressive disease course and are more likely to benefit from intensified systemic therapies such as triplet treatment (ADT, docetaxel, and an androgen receptor signaling inhibitor) [24]. For instance, the ARASENS trial, which evaluated these multimodal regimens, demonstrated the greatest survival benefit among patients with high-volume disease, while patients with low-volume disease did not derive the same degree of benefit [35]. Since the reporting of this trial, clinical guidelines have increasingly emphasized volume-based stratification to individualize therapeutic strategies [21,22]. As such, accurate disease volume assessment using sensitive imaging modalities like PSMA PET-CT is essential for identifying eligible candidates for intensified regimens.

## 3. Key Clinical Trials and Treatment Strategies

**Figure 1:**
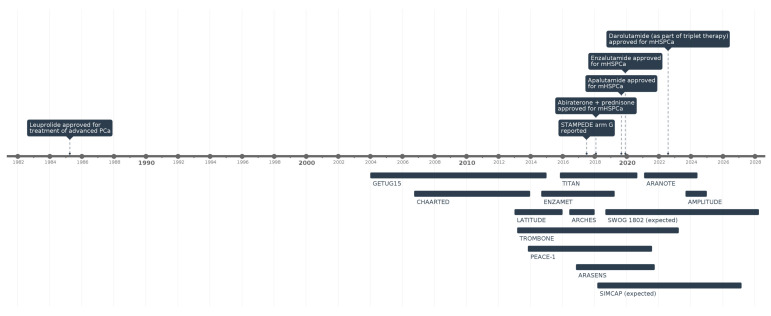
Timeline of key clinical trials and FDA drug approvals in mHSPCa.

While surgical castration has been a key treatment for mHSPCa since the 1940s, by the 1990s, medical castration, also known as androgen deprivation therapy (ADT), had become the cornerstone of treatment for men with mHSPCa (Figure 1). In the last decade, several clinical trials evaluating combination therapies based on novel hormonal agents and chemotherapy have demonstrated significant improvements in overall survival (OS) and progression, revolutionizing the therapeutic landscape. A comparison of major clinical trials of therapeutic options for mHSPCa can be seen in Table 1.

### 3.1. Doublet Therapy

Docetaxel was the first agent to show significant survival benefits when combined with ADT in metastatic CRPC [36]. In September 2015, the CHAARTED trial established that adding docetaxel to ADT improved OS by 17 months compared to ADT alone, with a more pronounced effect in patients with high-volume disease (HR 0.63, 95% CI 0.50–0.79) [32]. However, patients with low-volume disease failed to demonstrate a survival benefit (HR 1.04, 95% CI 0.70–1.55). These findings have suggested the need for tailored treatment strategies, likely due to the heterogeneity in tumor biology and pathophysiology that underlies high-volume and low-volume disease.

The STAMPEDE trial further expanded on these findings, demonstrating that docetaxel, when combined with ADT, consistently reduced the risk of death across all metastasis-burden subgroups (OS) HR 0.81, 95% CI 0.69–0.95, *p* = 0.009) [37]. The trial’s innovative multi-arm, multi-stage design allowed for the evaluation of multiple treatment strategies simultaneously. In contrast, the GETUG-15 trial, which also evaluated docetaxel with ADT and was published in 2016, did not achieve a statistically significant OS benefit, likely due to its smaller sample size and lower statistical power [38]. Nevertheless, the results of GETUG-15 suggested a trend favoring docetaxel, particularly in high-volume disease, prompting the authors to recommend discussing chemotherapy as a treatment option for these patients. Finally, a meta-analysis of CHAARTED and GETUG-15 further clarified the role of docetaxel, demonstrating significant survival benefits for patients with high-volume metastatic disease (HR 0.68, 95% CI 0.56–0.82, *p* < 0.001) [39]. Collectively, these trials established docetaxel as a standard addition to ADT in the treatment of high-volume mHSPCa and as an important consideration for selected patients with low-volume disease. Reflecting this evidence, the STAMPEDE trial adjusted its design after 2015, incorporating docetaxel into the standard-of-care control arm [40].

### 3.2. Abiraterone

Abiraterone acetate is a potent inhibitor of 17α-hydroxylase/C17,20-lyase, enzymes required for androgen biosynthesis [41]. Since its introduction, abiraterone has emerged as an important drug in the management of mHSPCa, particularly among patients exhibiting two or more high-risk features: visceral disease, a Gleason score of ≥8, or the presence of ≥3 lesions on a bone scan. Abiraterone is co-administered with prednisone to ameliorate potential adverse side effects of the medication. Trial results published in July of 2017 in the LATITUDE study were pivotal in establishing the efficacy of abiraterone. The addition of abiraterone and prednisone to ADT significantly improved OS (48.2 months vs. 36.5 months) and radiographic progression-free (rPFS) survival. This combination led to a 38% reduction in mortality compared to ADT alone [42,43]. Two years later, Arm G of the STAMPEDE trial provided additional evidence in support of using abiraterone in a broader population, including patients with both high- and low-risk mHSPCa. At a median follow-up of 40 months, the STAMPEDE trial demonstrated that abiraterone in combination with ADT demonstrated an OS benefit in a group of 1917 men. A 37% relative improvement in survival was noted with an HR of 0.63 (95% CI, 0.52–0.76, *p* < 0.0001) [44]. Beyond its survival benefits, abiraterone delayed the time to skeletal-related events and significantly improved quality of life [45]. These results make abiraterone an appealing option for patients who are unable to tolerate chemotherapy.

### 3.3. Enzalutamide

Building on the success of abiraterone as an androgen synthesis inhibitor, focus shifted to alternative strategies targeting the androgen signaling pathway. In this context, enzalutamide emerged as a second-generation androgen receptor signaling inhibitor (ARSI). Also published in early 2019, the ENZAMET trial randomized 1125 mHSPCa patients to receive ADT plus standard non-steroidal anti-androgen or ADT plus enzalutamide [46]. At three years, OS was 8% higher in the enzalutamide group when compared to the standard non-steroidal antiandrogen group, corresponding to an HR for death of 0.67 (95% CI 0.52–0.86, *p* = 0.002). Importantly, patients who did not receive concurrent docetaxel experienced the most pronounced OS benefit (HR 0.53, 95%CI 0.37–0.75), underscoring enzalutamide’s potential as an alternative to chemotherapy, especially among those who cannot tolerate the side effects of the taxane class of medications. By late 2019, the ARCHES trial confirmed the efficacy of enzalutamide, demonstrating a reduction in the risk of radiographic progression and death in the enzalutamide group among a cohort of 1150 men already receiving lifetime ADT [47].

### 3.4. Apalutamide

Apalutamide, another ARSI, has demonstrated its efficacy in the management of mHSPCa. The TITAN trial, published in July 2019, evaluated the addition of apalutamide to ADT and showed significant improvements in both OS and progression-free survival (PFS) compared to ADT alone [48]. At 24 months, the OS rate was 82.4% in the apalutamide group versus 73.5% with ADT alone, corresponding to an HR for death of 0.67 (95% CI, 0.51–0.89; *p* = 0.005). Importantly, the benefits of apalutamide were observed across all subgroups, suggesting its high capability regardless of disease volume at treatment initiation. In addition to clear survival improvement, patients who received apalutamide maintained their baseline health-related quality of life. Apalutamide has demonstrated a favorable side-effect profile, making it an appealing option for individuals who may be more vulnerable to the toxicities of chemotherapy. Common adverse effects observed more often in the apalutamide arm were rash and hypothyroidism.

### 3.5. Triplet Therapy

Building on the success of doublet therapy and the emergence of novel antiandrogen agents, recent research has focused on exploring combination strategies to further enhance treatment outcomes. Triplet therapy, combining ADT, docetaxel, and ARSI, has emerged as a promising approach in the treatment of mHSPCa. The results of the ARASENS (published in May 2022) and PEACE-1 (published in December 2021) trials have cemented the viability of this treatment strategy. The ARASENS study was conducted as a phase 3 trial of 1306 men with mHSPCa which evaluated the addition of darolutamide, another ARSI, to traditional doublet therapy, and demonstrated a marked improvement in OS at four years (62.7% vs. 50.4%) compared to docetaxel plus ADT alone [49]. Adverse events were similar in the two groups, with incidences of most common adverse events occurring during the overlapping docetaxel treatment period in both groups. Notably, results from the ARANOTE trial, published in 2024, suggested that darolutamide may be efficacious within the context of triplet therapy. While this phase 3 study failed to identify a statistically significant reduction in the risk of death with darolutamide plus ADT compared to ADT alone (HR 0.81, 95% CI 0.59–1.12), it did observe a significantly lower risk of rPFS (HR 0.54, 95% CI 0.41–0.71, *p* < 0.001) [50]. Similar to ARASENS, PEACE-1 investigated the addition of abiraterone to ADT and docetaxel, showing significant benefits for OS (HR 0.82, 95.1% CI 0.69–0.98, *p* < 0.001) and rPFS (HR 0.54, 99.9% CI 0.41–0.71, *p* < 0.001), particularly in patients with high-volume disease [51]. These results underline the importance of intensifying initial treatment for select patients to maximize long-term survival benefits.

### 3.6. Radiation Therapy

Radiation therapy to the primary tumor, combined with ADT, has been investigated as a treatment option for mHSPCa in several key trials. The STAMPEDE trial (arm H) and the HORRAD trial specifically evaluated the addition of external beam radiotherapy (EBRT) to ADT versus ADT alone in men with metastatic prostate cancer. Within the STAMPEDE trial, which included 2061 men with newly diagnosed metastatic disease, a 2020 secondary subgroup analysis revealed that men with low-volume metastatic disease experienced a significant improvement in three-year survival with the addition of radiotherapy (81% vs. 73%, HR 0.68, 95% CI 0.52–0.90, *p* = 0.007) [40]. Radiotherapy was also associated with improved failure-free survival across all patients, including those with both low-volume and high-volume disease (HR 0.76, 95% CI 0.68–0.84, *p* < 0.001). However, no OS benefit was observed across all patients regardless of disease burden, suggesting a targeted benefit for low-volume disease.

The HORRAD trial, reported in 2018, which randomized 432 men with PSA > 20 ng/mL and primary bone metastases to receive ADT with or without EBRT, similarly observed mixed results with the addition of radiotherapy [52]. While there was no statistically significant difference in OS between the EBRT plus ADT group (45 months) and the ADT-alone group (43 months, *p* = 0.4), a reduction in PSA progression was noted in the radiotherapy group (HR 0.78; 95% CI 0.63–0.97, *p* = 0.02). Consequently, it is thought that the addition of radiotherapy could provide benefits in certain patient populations, but the selection criteria remain unclear.

In addition to its contributions to advancing triplet therapy, the PEACE-1 trial offered further insights into the role of radiotherapy in combination with systemic treatments [51]. PEACE-1 implemented a 2 × 2 factorial design randomly assigning 1172 men to receive standard of care, standard of care plus radiotherapy, standard of care plus abiraterone, and standard of care plus abiraterone and radiotherapy. At a median follow-up of 6.9 years, the trial concluded that the addition of local radiotherapy to any treatment regimen did not result in an improvement in OS (HR 0.98, 95% CI 0.74–1.28, *p* = 0.86).

In 2021, the STOPCAP systematic review and meta-analysis, incorporating data from the STAMPEDE, HORRAD, and PEACE-1 trials, concluded that radiotherapy does not universally improve OS when added to ADT in mHSPCa [53]. However, the analysis highlighted significant benefits in biochemical PFS and FFS, particularly among men with low metastatic burden. Taken together, the available clinical evidence suggests that radiotherapy to the primary tumor should not be considered an effective option in men with metastatic PCa (Figure 1).

**Table 1:** Comparison of clinical trials for therapeutic options in the management of mHSPCa.

Therapy	Trial	Mechanism of Action	N	Inclusion Criteria	HR for Death	Progression-Free Survival HR	Adverse Events
Docetaxel + ADT vs. ADT alone [32]	CHAARTED	Chemohormonal therapy	790	Radiologic evidence of metastatic disease	Overall cohort: 0.72 (0.59–0.89)	*C: 0.62 (0.51–0.75)	Neutropenia, febrile neutropenia
Docetaxel + ADT vs. ADT alone [37]	STAMPEDE ARM C	Chemohormonal therapy	2962	mPCa patients initiating hormone therapy	Overall cohort: 0.78 (0.66–0.93)	*C: 0.61 (0.53–0.71)	Neutropenia, febrile neutropenia
Docetaxel + ADT vs. ADT alone [38]	GETUG-15	Chemohormonal therapy	385	Patients with metastatic disease	Overall cohort: ** 1.01 (0.75–1.36)	*R: 0.75 (0.58–0.97)	Neutropenia
Abiraterone + prednisone + ADT vs. ADT alone [42,54]	LATITUDE	Androgen biosynthesis inhibitor	1199	High-risk mHSPCa	Overall cohort: 0.66 (0.56–0.78)	*R: 0.45 (0.40–0.51)	Hypertension, hypokalemia
Abiraterone + ADT vs. ADT alone [44]	STAMPEDE ARM G	Androgen biosynthesis inhibitor	1917	mPCa patients initiating hormone therapy	Overall cohort: 0.63 (0.52–0.76)	*C: 0.29 (0.25–0.34)	Hypertension, respiratory disorders
Enzalutamide + ADT vs. NSAA SOC [46]	ENZAMET	Androgen receptor signaling inhibitor	1125	Patients with metastatic disease	Overall cohort: 0.70 (0.58–0.84)	*B: 0.39 (0.33–0.47)	Fatigue, seizures
Enzalutamide + ADT vs. ADT alone [47]	ARCHES	Androgen receptor signaling inhibitor	1150	Patients with metastatic disease	Overall cohort: 0.66 (0.53–0.81)	*R: 0.39 (0.30–0.50)	Fatigue, hypertension
Apalutamide + ADT vs. ADT alone [48]	TITAN	Androgen receptor signaling inhibitor	1052	Patients with metastatic disease	Overall cohort: 0.52 (0.42–0.64)	*R: 0.48 (0.39–0.60)	Rash, hypothyroidism
Darolutamide + Docetaxel + ADT vs. docetaxel + ADT [35,49]	ARASENS	Multimodal	1306	Candidates for doublet therapy	High-volume cohort: 0.69 (0.57–0.82)	*C: 0.36 (0.30–0.42)	Neutropenia
Darolutamide + ADT vs. ADT [50]	ARANOTE	Androgen receptor signaling inhibitor	669	Patients with metastatic disease	Overall cohort: ** 0.81 (0.59–1.12)	*R: 0.54 (0.41–0.71)	Fatigue
Abiraterone + docetaxel + ADT vs. docetaxel + ADT [51]	PEACE-1	Multimodal	1173	De novo mHSPCa with high metastatic burden	Overall cohort: 0.82 (0.69–0.98)	*R: 0.54 (0.41–0.71)	Neutropenia, hypertension
EBRT + ADT + docetaxel vs. ADT + docetaxel [40]	STAMPEDE ARM H	Radiation therapy	2061	mPCa patients initiating hormone therapy	Overall cohort: ** 0.92 (0.80–1.06)	** 0.96 (0.85–1.08)	Urinary incontinence
EBRT + ADT vs. ADT alone	HORRAD	Radiation therapy	432	Newly diagnosed mHSPCa with PSA > 20	Overall cohort: ** 0.9 (0.70–1.14)	B: 0.78 (0.63–0.97)	Bowel and urinary symptoms
EBRT + ADT	STOPCAP	Radiation therapy	N/A	N/A	Overall cohort: ** 0.92 (0.81–1.04)	B: 0.74 (0.67–0.82)	N/A

*R—radiographic progression-free survival; *B—biochemical progression-free survival; *C—clinical progression-free survival. **—no statistically significant difference.

## 4. Personalized Treatment Considerations and Patient Selection

Despite the high utility of recently discovered and approved therapies for mHSPCa, there is limited head-to-head data comparing efficacy and adverse event profiles among therapeutic options, restricting the development of definitive treatment algorithms [21]. Understanding the therapeutic benefits as well as the distinct side-effect profiles and contraindications of available therapies is essential for optimizing patient care.

Docetaxel exhibits a similar adverse effect profile to medications within the taxane class, including peripheral neuropathy and myalgias [55]. In the CHAARTED trial, docetaxel was associated with a 2% increase in grade 3–4 hypersensitivity reactions and infections with neutropenia, a 4% increase in grade 3 fatigue, and a 1% or lower incidence in stomatitis, grade 3 diarrhea, and neuropathies compared to placebo [55]. The most clinically significant adverse event was febrile neutropenia, with incidence rates of 6% in CHAARTED and 8% and 12% in the GETUG-AFU15 and STAMPEDE trials, respectively [56]. Consequently, docetaxel should not be prescribed in patients with concurrent neutropenia, significant neuropathies or a history of severe hypersensitivity reactions.

Abiraterone has been linked to hepatotoxicity, as well as increased rates of hypokalemia (9%) and hypertension (10%) in the LATITUDE trial [42,57]. As such, patients with Child-Pugh Class C hepatic impairment should be carefully monitored if abiraterone is used. The likely mechanism by which abiraterone causes hypertension is through the accumulation of excess mineralocorticoids. Thus, concurrent hypokalemia or uncontrolled hypertension are relative contraindications and should be optimized prior to abiraterone therapy.

ARSIs—including apalutamide, enzalutamide, and darolutamide—share common adverse effects associated with ADT such as hot flashes, fatigue, anemia, osteoporosis, and weight gain [58,59]. Enzalutamide, evaluated in the PREVAIL trial, demonstrated particularly high rates of hot flash episodes (17%), hypertension (57%), and falls (22%), with 7% of patients experiencing grade 3+ hypertensive events [59]. Notably, seizure risks observed in the AFFIRM, PREVAIL, and TITAN trials have led to the addition of warning labels by the FDA for both enzalutamide and apalutamide [60]. Within this class, enzalutamide is considered the least safe due to its pronounced seizure and hypertensive risk profile. The TITAN trial identified increased rates of rash (18.6%), hypothyroidism (5.4%), and ischemic heart disease (2.9%) with apalutamide treatment [48]. In contrast, darolutamide demonstrated a more favorable safety profile in the ARASENS trial, with only modest increases in hypertension (4.5%) and rash (3%) [49]. This improved tolerability is likely due to darolutamide’s limited capacity to cross the blood–brain barrier, but findings from the ARANOTE trial suggest this may come at the cost of reduced efficacy, as the addition of darolutamide did not improve OS [50,58]. Meanwhile, radiotherapy, as evaluated in the STAMPEDE trial with a median follow-up of 37 months (IQR 24–48 months), exhibited a comparatively mild side-effect profile, with less than a 1% increase in proctitis, cystitis, hematuria, and urethral stricture [40]. However, recent evidence suggests that prostate radiotherapy may increase the long-term risk of secondary rectal and bladder cancers, which are associated with worse survival outcomes compared to their primary counterparts [61,62].

Given the current uncertainty regarding the comparative efficacy of different mHSPCa treatment combinations, treatment decisions should be individualized, based on patient goals of care, drug tolerances, comorbidities, economic considerations, and clinician expertise. Recent studies have examined factors associated with the choice of mHPSCa management recommendations. Among these, physician comfort, familiarity with new therapeutics, and specialty are associated with prescription patterns. For example, a retrospective analysis of Medicare payor data demonstrated that urologists more frequently opted to prescribe enzalutamide over abiraterone/prednisone, which was more frequently used by medical oncologists [63]. Understanding variation between the uptake and utilization of novel therapeutics will be crucial to more effective implementation, particularly within communities that experience disparate access to care [64,65]. Regardless, once a personalized treatment plan is established, both physicians and patients must remain flexible and be prepared to pivot swiftly if necessary, recognizing the urgency of effectively managing mHSPCa.

## 5. Emerging Therapies and Future Research in mHSPCa

The treatment landscape for mHSPCa is evolving rapidly, with numerous promising ongoing clinical trials investigating novel therapeutic combinations. One area of focus is the combination of targeted therapies with traditional treatments to improve patient outcomes for those with alterations in protein signaling pathways. In particular, the PI3K-Akt-mTOR pathway has been identified as critical to the development and progression of PCa [66]. In fact, evidence suggests that patients with more advanced stage or castration-resistant disease are more likely to present with alterations, causing the pathway to be constitutively active [67,68]. One ongoing trial is exploring the combination of standard doublet therapy with the addition of small molecule protein kinase B (Akt) inhibitors, such as capivasertib in men with PTEN-deficient mHSPCa tumors [69]. Currently, there are no approved AKT inhibitors for mHSPCa.

The use of poly (ADP-ribose) polymerase inhibitors (PARPi) is another burgeoning area of research in the treatment of PCa. Olaparib, rucaparib, and talzoparib were first studied for clinical use among patients with CRPC in the PROfound, TRITON, and TALAPRO trials, respectively. These drugs were shown to improve OS for men with homologous recombination repair deficiencies and thus achieved FDA approval by 2023. EvoPAR-Prostate01 is a Phase III, randomized, controlled trial assessing the utility of saruparib, another PARPi, among mHSPCa patients with and without homologous recombination repair (HRR) mutations [70]. Similarly, niraparib, another PARPi, is being evaluated in the AMPLITUDE trial in conjunction with prednisone and abiraterone for patients with mHSPCa who have germline or somatic HRR mutations [71]. The results of the AMPLITUDE trail are expected to be reported in 2025.

Furthermore, due to the success of triplet therapy demonstrated in ARASENS and PEACE-1, rezvilutamide, a new ARSI, was evaluated in the CHART phase 3 trial. At second interim analysis, rezvilutamide and ADT significantly improved OS when compared to bicalutamide and ADT (HR 0.58, 95%CI 0.33–0.58, *p* < 0.001) [72]. The optimal duration of treatment is being evaluated in a randomized, factorial assignment clinical trial which began recruitment in June 2023 [73]. This study also aims to assess differences in rPFS at 6-, 12-, 18-, and 24-month treatment intervals.

Cytoreductive radical prostatectomy (cRP) alongside systemic therapy is also an area of active investigation. In other genitourinary malignancies, especially renal cell carcinoma, the addition of cytoreductive surgery has demonstrated benefits for progression and survival [74]. The rationale for local treatment of the primary tumor is that by eliminating the primary tumor, patients can effectively achieve local symptom control and reduce metastasis spread, thus enhancing the efficacy of systemic treatment [33]. The TRoMbone clinical trial first established that the randomization of cRP with systemic therapy could be feasible with no substantial impact on quality of life [34]. Evidence regarding optimal patient selection for cytoreductive radical prostatectomy is mixed. Retrospective analyses have generally identified patients with low-volume metastatic disease as the most likely to benefit from cRP [75,76]. In contrast, population-based studies have reported survival advantages associated with cRP irrespective of metastatic burden [77,78].

Two trials aiming to evaluate combining cRP with standard-of-care therapy are underway [79,80]. SIMCAP, conducted within the United States and Asia Pacific, is a randomized phase 2.5 trial which began in 2018 and is expected to report on differences in failure-free survival in 2027. The SWOG 1802 clinical trial, which also began international recruitment in 2018, is expected to be the first study to provide phase 3 evidence evaluating the efficacy of cRP, particularly in terms of OS. Simultaneously, another phase III clinical trial, conducted in China, began patient recruitment in 2016 and is expected to report on rPFS and the safety of the intervention arm by 2028.

Immune checkpoint inhibitors, including those that target programmed cell death protein 1 (PD-1) have become a key management option for patients with advanced bladder and kidney cancer. The efficacy of these immunotherapeutics for high-volume mHSPCa are actively evaluated in the PROSTRATEGY trial, which aims to combine immune checkpoint inhibitors, including nivolumab and ipilimumab, with traditional doublet therapy in patients with high-volume mHSPCa [81]. Given the success of immunotherapy in other genitourinary malignancies, their inclusion in clinical trials for PCa is promising and may present possibilities for new treatment combinations in mHSPCa.

The management of mHSPCa is increasingly incorporating biomarker-driven therapies to enhance treatment precision. Liquid biopsies, which identify blood-based abnormalities like circulating tumor DNA (ctDNA) and circulating tumor cells (CTCs), are a promising option to dynamically assess tumor characteristics. Detection of AR-V7 variants in CTCs, for instance, has been associated with tumor resistance to ARSIs [82]. Additionally, targeted sequencing of ctDNA can better identify candidates for PARPi therapy [83]. The ProBio trial is a biomarker-driven platform trial aimed at personalizing treatment for PCa [84]. In this study, patients are randomized to either a control arm (standard care) or an experimental arm based on biomarker signatures from tissue or liquid biopsies. Using an adaptive Bayesian framework and a sequential multiple assignment trial (SMART) approach, the trial continuously adjusts randomization to identify the most effective therapies, offering a personalized strategy to improve outcomes in prostate cancer treatment.

## 6. Conclusions

The treatment landscape for mHSPCa has evolved significantly, with combination therapies, including ADT plus ARSIs or chemotherapy, now being the standard of care. Recent advancements, such as triplet therapy, biomarker driven treatments, and emerging targeted agents, offer promising strategies to further improve outcomes. Precision medicine, guided by genomic profiling and advanced imaging, is shaping a more personalized approach to treatment. As ongoing clinical trials continue to refine therapeutic strategies, optimizing treatment selection based on disease burden, molecular characteristics, and patient factors remains critical in delaying disease progression and improving survival.

## Data Availability

This review did not generate or analyze any original data. All data discussed are from previously published sources, which are cited appropriately within the manuscript. No new data were created or analyzed in this study.

## Abbreviations

The following abbreviations are used in this manuscript:

mHSPCa	Metastatic Hormone-Sensitive Prostate Cancer
CRPC	Castration-Resistant Prostate Cancer
PCa	Prostate Cancer
ADT	Androgen Deprivation Therapy
ARSI	Androgen Receptor Signaling Inhibitor
OS	Overall Survival
rPFS	Radiographic Progression-Free Survival
PFS	Progression-Free Survival
PSA	Prostate-Specific Antigen
USPSTF	United States Preventive Services Task Force
AUA	American Urological Association
NCCN	National Comprehensive Cancer Network
CT	Computer Tomography
MRI	Magnetic Resonance Imaging
PSMA PET-CT	Prostate-Specific Membrane Antigen Positron Emission Tomography-Compute Tomography
EBRT	External Beam Radiotherapy
SNP	Single Nucleotide Polymorphism
HR	Hazard Ratio
FDA	Food and Drug Administration
PD-1	Programmed Cell Death Protein 1
PARPi	Poly (ADP-ribose) Polymerase Inhibitor
HRR	Homologous Recombination Repair
SMART	Sequential Multiple Assignment Randomized Trial
cRP	Cytoreductive radical prostatectomy

## References

[1] Siegel R.L., Giaquinto A.N., Jemal A. (2024). Cancer statistics, 2024. CA Cancer J. Clin..

[2] Siegel R.L., Kratzer T.B., Giaquinto A.N., Sung H., Jemal A. (2025). Cancer statistics, 2025. CA Cancer J. Clin..

[3] Borregales L.D., DeMeo G., Gu X., Cheng E., Dudley V., Schaeffer E.M., Nagar H., Carlsson S., Vickers A., Hu J.C. (2022). Grade Migration of Prostate Cancer in the United States During the Last Decade. JNCI J. Natl. Cancer Inst..

[4] Jemal A., Culp M.B., Ma J., Islami F., A Fedewa S. (2021). Prostate Cancer Incidence 5 Years After US Preventive Services Task Force Recommendations Against Screening. JNCI J. Natl. Cancer Inst..

[5] Zhang A.C., Rasul R., Golden A., Feuerstein M.A. (2021). Incidence and mortality trends of metastatic prostate cancer: Surveillance, Epidemiology, and End Results database analysis. Can. Urol. Assoc. J..

[6] Schafer E.J., Jemal A., Wiese D., Sung H., Kratzer T.B., Islami F., Dahut W.L., Knudsen K.E. (2023). Disparities and Trends in Genitourinary Cancer Incidence and Mortality in the USA. Eur. Urol..

[7] Kelly S.P., Anderson W.F., Rosenberg P.S., Cook M.B. (2018). Past, Current, and Future Incidence Rates and Burden of Metastatic Prostate Cancer in the United States. Eur. Urol. Focus.

[8] Moyer V.A., US Preventive Services Task Force (2012). Screening for prostate cancer: US Preventive Services Task Force recommendation statement. Ann. Intern. Med..

[9] Leapman M.S., Wang R., Park H., Yu J.B., Sprenkle P.C., Cooperberg M.R., Gross C.P., Ma X. (2022). Changes in Prostate-Specific Antigen Testing Relative to the Revised US Preventive Services Task Force Recommendation on Prostate Cancer Screening. JAMA Oncol..

[10] Siegel D.A., Elizabeth O.M., Richards T.B., Dowling N.F., Weir H.K. (2020). Prostate cancer incidence and survival, by stage and race/ethnicity—United States, 2001–2017. MMWR. Morb. Mortal. Wkly. Rep..

[11] Wenzel M., Preisser F., Hoeh B., Schroeder M., Würnschimmel C., Steuber T., Heinzer H., Banek S., Ahrens M., Becker A. (2021). Impact of Time to Castration Resistance on Survival in Metastatic Hormone Sensitive Prostate Cancer Patients in the Era of Combination Therapies. Front. Oncol..

[12] Freedland S.J., Davis M., Epstein A.J., Arondekar B., Ivanova J.I. (2024). Real-world treatment patterns and overall survival among men with Metastatic Castration-Resistant Prostate Cancer (mCRPC) in the US Medicare population. Prostate Cancer Prostatic Dis..

[13] Djavan B., Eastham J., Gomella L., Tombal B., Taneja S., Dianat S.S., Kazzazi A., Shore N., Abrahamsson P., Cheetham P. (2012). Testosterone in prostate cancer: The Bethesda consensus. BJU Int..

[14] Klotz L., Breau R.H., Collins L.L., Gleave M.E., Pickles T., Pouliot F., Saad F. (2017). Maximal testosterone suppression in the management of recurrent and metastatic prostate cancer. Can. Urol. Assoc. J..

[15] Pernar C.H., Ebot E.M., Wilson K.M., Mucci L.A. (2018). The epidemiology of prostate cancer. Cold Spring Harb. Perspect. Medicine..

[16] Na R., Zheng S.L., Han M., Yu H., Jiang D., Shah S., Ewing C.M., Zhang L., Novakovic K., Petkewicz J. (2017). Germline mutations in ATM and BRCA1/2 distinguish risk for lethal and indolent prostate cancer and are associated with early age at death. Eur. Urol..

[17] Ewing C.M., Ray A.M., Lange E.M., Zuhlke K.A., Robbins C.M., Tembe W.D., Wiley K.E., Isaacs S.D., Johng D., Wang Y. (2012). Germline Mutations in *HOXB13* and Prostate-Cancer Risk. N. Engl. J. Med..

[18] Bergengren O., Pekala K.R., Matsoukas K., Fainberg J., Mungovan S.F., Bratt O., Bray F., Brawley O., Luckenbaugh A.N., Mucci L. (2023). 2022 Update on Prostate Cancer Epidemiology and Risk Factors—A Systematic Review. Eur. Urol..

[19] Shiota M., Nemoto S., Ikegami R., Tatarano S., Kamoto T., Kobayashi K., Sakai H., Igawa T., Kamba T., Fujimoto N. (2024). Predictive model of castration resistance in advanced prostate cancer by machine learning using genetic and clinical data: KYUCOG-1401-A study. BJC Rep..

[20] Finelli A., Beer T.M., Chowdhury S., Evans C.P., Fizazi K., Higano C.S., Kim J., Martin L., Saad F., Saarela O. (2021). Comparison of joint and landmark modeling for predicting cancer progression in men with castration-resistant prostate cancer: A secondary post hoc analysis of the PREVAIL randomized clinical trial. JAMA Netw. Open.

[21] Lowrance W., Dreicer R., Jarrard D.F., Scarpato K.R., Kim S.K., Kirkby E., Buckley D.I., Griffin J.C., Cookson M.S. (2023). Updates to Advanced Prostate Cancer: AUA/SUO Guideline (2023). J. Urol..

[22] Schaeffer E.M., Srinivas S., Adra N., An Y., Bitting R., Chapin B., Cheng H.H., D’Amico A.V., Desai N., Dorff T. (2024). NCCN Guidelines® Insights: Prostate Cancer, Version 3.2024: Featured Updates to the NCCN Guidelines. J. Natl. Compr. Cancer Netw..

[23] Lowrance W.T., Breau R.H., Chou R., Chapin B.F., Crispino T., Dreicer R., Jarrard D.F., Kibel A.S., Morgan T.M., Morgans A.K. (2021). Advanced Prostate Cancer: AUA/ASTRO/SUO Guideline PART I. J. Urol..

[24] Schaeffer E.M., Srinivas S., Adra N., An Y., Barocas D., Bitting R., Bryce A., Chapin B., Cheng H.H., D’Amico A.V. (2023). Prostate Cancer, Version 4.2023, NCCN Clinical Practice Guidelines in Oncology. J. Natl. Compr. Cancer Netw. JNCCN.

[25] Dyrberg E., Hendel H.W., Huynh T.H.V., Klausen T.W., Løgager V.B., Madsen C., Pedersen E.M., Pedersen M., Thomsen H.S. (2018). 68Ga-PSMA-PET/CT in comparison with 18F-fluoride-PET/CT and whole-body MRI for the detection of bone metastases in patients with prostate cancer: A prospective diagnostic accuracy study. Eur. Radiol..

[26] Hofman M.S., Lawrentschuk N., Francis R.J., Tang C., Vela I., Thomas P., Rutherford N., Martin J.M., Frydenberg M., Shakher R. (2020). Prostate-specific membrane antigen PET-CT in patients with high-risk prostate cancer before curative-intent surgery or radiotherapy (proPSMA): A prospective, randomised, multicentre study. Lancet.

[27] Zhou J., Gou Z., Wu R., Yuan Y., Yu G., Zhao Y. (2019). Comparison of PSMA-PET/CT, choline-PET/CT, NaF-PET/CT, MRI, and bone scintigraphy in the diagnosis of bone metastases in patients with prostate cancer: A systematic review and meta-analysis. Skelet. Radiol..

[28] Sonni I., Felker E.R., Lenis A.T., Sisk A.E., Bahri S., Allen-Auerbach M.S., Armstrong W.R., Suvannarerg V., Tubtawee T., Grogan T. (2022). Head-to-Head Comparison of ^68^Ga-PSMA-11 PET/CT and mpMRI with a Histopathology Gold Standard in the Detection, Intraprostatic Localization, and Determination of Local Extension of Primary Prostate Cancer: Results from a Prospective Single-Center Imaging Trial. J. Nucl. Med..

[29] Liu F., Dong J., Shen Y., Yun C., Wang R., Wang G., Tan J., Wang T., Yao Q., Wang B. (2021). Comparison of PET/CT and MRI in the Diagnosis of Bone Metastasis in Prostate Cancer Patients: A Network Analysis of Diagnostic Studies. Front. Oncol..

[30] Lokeshwar S.D., Choksi A.U., Haltstuch D., Rahman S.N., Press B.H., Syed J., Hurwitz M.E., Kim I.Y., Leapman M.S. (2023). Personalizing approaches to the management of metastatic hormone sensitive prostate cancer: Role of advanced imaging, genetics and therapeutics. World J. Urol..

[31] Van Den Bergh L., Lerut E., Haustermans K., Deroose C.M., Oyen R., Isebaert S., Budiharto T., Ameye F., Mottaghy F.M., Bogaerts K. (2015). Final analysis of a prospective trial on functional imaging for nodal staging in patients with prostate cancer at high risk for lymph node involvement. Urol. Oncol. Semin. Orig. Investig..

[32] Kyriakopoulos C.E., Chen Y.-H., Carducci M.A., Liu G., Jarrard D.F., Hahn N.M., Shevrin D.H., Dreicer R., Hussain M., Eisenberger M. (2018). Chemohormonal Therapy in Metastatic Hormone-Sensitive Prostate Cancer: Long-Term Survival Analysis of the Randomized Phase III E3805 CHAARTED Trial. J. Clin. Oncol..

[33] Ranasinghe W., Chapin B.F., Kim I.Y., Sooriakumaran P., Lawrentschuk N. (2020). The cytoreductive prostatectomy in metastatic prostate cancer: What the individual trials are hoping to answer. BJU Int..

[34] Sooriakumaran P., Wilson C., Rombach I., Hassanali N., Aning J., Lamb A.D., Cathcart P., Eden C., Ahmad I., Rajan P. (2022). Feasibility and safety of radical prostatectomy for oligo-metastatic prostate cancer: The Testing Radical prostatectomy in men with prostate cancer and oligo-Metastases to the bone (TRoMbone) trial. BJU Int..

[35] Hussain M., Tombal B., Saad F., Fizazi K., Sternberg C.N., Crawford E.D., Shore N., Kopyltsov E., Kalebasty A.R., Bögemann M. (2023). Darolutamide Plus Androgen-Deprivation Therapy and Docetaxel in Metastatic Hormone-Sensitive Prostate Cancer by Disease Volume and Risk Subgroups in the Phase III ARASENS Trial. J. Clin. Oncol..

[36] Tannock I.F., De Wit R., Berry W.R., Horti J., Pluzanska A., Chi K.N., Oudard S., Théodore C., James N.D., Turesson I. (2004). Docetaxel plus Prednisone or Mitoxantrone plus Prednisone for Advanced Prostate Cancer. N. Engl. J. Med..

[37] James N.D., Sydes M.R., Clarke N.W., Mason M.D., Dearnaley D.P., Spears M.R., Ritchie A.W.S., Parker C.C., Russell J.M., Attard G. (2016). Addition of docetaxel, zoledronic acid, or both to first-line long-term hormone therapy in prostate cancer (STAMPEDE): Survival results from an adaptive, multiarm, multistage, platform randomised controlled trial. Lancet.

[38] Gravis G., Fizazi K., Joly F., Oudard S., Priou F., Esterni B., Latorzeff I., Delva R., Krakowski I., Laguerre B. (2013). Androgen-deprivation therapy alone or with docetaxel in non-castrate metastatic prostate cancer (GETUG-AFU 15): A randomised, open-label, phase 3 trial. Lancet Oncol..

[39] Gravis G., Boher J.-M., Chen Y.-H., Liu G., Fizazi K., Carducci M.A., Oudard S., Joly F., Jarrard D.M., Soulie M. (2018). Burden of Metastatic Castrate Naive Prostate Cancer Patients, to Identify Men More Likely to Benefit from Early Docetaxel: Further Analyses of CHAARTED and GETUG-AFU15 Studies. Eur. Urol..

[40] Parker C.C., James N.D., Brawley C.D., Clarke N.W., Hoyle A.P., Ali A., Ritchie A.W.S., Attard G., Chowdhury S., Cross W. (2018). Radiotherapy to the primary tumour for newly diagnosed, metastatic prostate cancer (STAMPEDE): A randomised controlled phase 3 trial. Lancet.

[41] De Bono J.S., Logothetis C.J., Molina A., Fizazi K., North S., Chu L., Chi K.N., Jones R.J., Goodman O.B., Saad F. (2011). Abiraterone and Increased Survival in Metastatic Prostate Cancer. N. Engl. J. Med..

[42] Fizazi K., Tran N., Fein L., Matsubara N., Rodriguez-Antolin A., Alekseev B.Y., Özgüroğlu M., Ye D., Feyerabend S., Protheroe A. (2017). Abiraterone plus Prednisone in Metastatic, Castration-Sensitive Prostate Cancer. N. Engl. J. Med..

[43] Fizazi K., Shore N., Tammela T.L., Ulys A., Vjaters E., Polyakov S., Jievaltas M., Luz M., Alekseev B., Kuss I. (2019). Darolutamide in nonmetastatic, castration-resistant prostate cancer. N. Engl. J. Med..

[44] James N.D., De Bono J.S., Spears M.R., Clarke N.W., Mason M.D., Dearnaley D.P., Ritchie A.W.S., Amos C.L., Gilson C., Jones R.J. (2017). Abiraterone for Prostate Cancer Not Previously Treated with Hormone Therapy. N. Engl. J. Med..

[45] Rush H.L., Murphy L., Morgans A.K., Clarke N.W., Cook A.D., Attard G., Macnair A., Dearnaley D.P., Parker C.C., Russell J.M. (2022). Quality of Life in Men with Prostate Cancer Randomly Allocated to Receive Docetaxel or Abiraterone in the STAMPEDE Trial. J. Clin. Oncol..

[46] Davis I.D., Martin A.J., Stockler M.R., Begbie S., Chi K.N., Chowdhury S., Coskinas X., Frydenberg M., Hague W.E., Horvath L.G. (2019). Enzalutamide with Standard First-Line Therapy in Metastatic Prostate Cancer. N. Engl. J. Med..

[47] Armstrong A.J., Szmulewitz R.Z., Petrylak D.P., Holzbeierlein J., Villers A., Azad A., Alcaraz A., Alekseev B., Iguchi T., Shore N.D. (2019). ARCHES: A Randomized, Phase III Study of Androgen Deprivation Therapy with Enzalutamide or Placebo in Men with Metastatic Hormone-Sensitive Prostate Cancer. J. Clin. Oncol..

[48] Chi K.N., Agarwal N., Bjartell A., Chung B.H., Pereira de Santana Gomes A.J., Given R., Juárez Soto Á., Merseburger A.S., Özgüroğlu M., Uemura H. (2019). Apalutamide for metastatic, castration-sensitive prostate cancer. N. Engl. J. Med..

[49] Smith M.R., Hussain M., Saad F., Fizazi K., Sternberg C.N., Crawford E.D., Kopyltsov E., Park C.H., Alekseev B., Montesa-Pino Á. (2022). Darolutamide and survival in metastatic, hormone-sensitive prostate cancer. N. Engl. J. Med..

[50] Saad F., Vjaters E., Shore N., Olmos D., Xing N., Gomes A.J.P.d.S., Mota A.C.d.A., Salman P., Jievaltas M., Ulys A. (2024). Darolutamide in Combination with Androgen-Deprivation Therapy in Patients with Metastatic Hormone-Sensitive Prostate Cancer From the Phase III ARANOTE Trial. J. Clin. Oncol..

[51] Fizazi K., Foulon S., Carles J., Roubaud G., McDermott R., Fléchon A., Tombal B., Supiot S., Berthold D., Ronchin P. (2022). Abiraterone plus prednisone added to androgen deprivation therapy and docetaxel in de novo metastatic castration-sensitive prostate cancer (PEACE-1): A multicentre, open-label, randomised, phase 3 study with a 2 × 2 factorial design. Lancet.

[52] Boevé L.M., Hulshof M.C., Vis A.N., Zwinderman A.H., Twisk J.W., Witjes W.P., Delaere K.P., van Moorselaar R.J.A., Verhagen P.C., van Andel G. (2019). Effect on Survival of Androgen Deprivation Therapy Alone Compared to Androgen Deprivation Therapy Combined with Concurrent Radiation Therapy to the Prostate in Patients with Primary Bone Metastatic Prostate Cancer in a Prospective Randomised Clinical Trial: Data from the HORRAD Trial. Eur. Urol..

[53] Burdett S., Boevé L.M., Ingleby F.C., Fisher D.J., Rydzewska L.H., Vale C.L., van Andel G., Clarke N.W., Hulshof M.C., James N.D. (2019). Prostate Radiotherapy for Metastatic Hormone-sensitive Prostate Cancer: A STOPCAP Systematic Review and Meta-analysis. Eur. Urol..

[54] Rydzewska L.H., Burdett S., Vale C.L., Clarke N.W., Fizazi K., Kheoh T., Mason M.D., Miladinovic B., James N.D., Parmar M.K. (2017). Adding abiraterone to androgen deprivation therapy in men with metastatic hormone-sensitive prostate cancer: A systematic review and meta-analysis. Eur. J. Cancer.

[55] Sweeney C.J., Chen Y.-H., Carducci M., Liu G., Jarrard D.F., Eisenberger M., Wong Y.-N., Hahn N., Kohli M., Cooney M.M. (2015). Chemohormonal Therapy in Metastatic Hormone-Sensitive Prostate Cancer. N. Engl. J. Med..

[56] Tucci M., Bertaglia V., Vignani F., Buttigliero C., Fiori C., Porpiglia F., Scagliotti G.V., Di Maio M. (2016). Addition of Docetaxel to Androgen Deprivation Therapy for Patients with Hormone-sensitive Metastatic Prostate Cancer: A Systematic Review and Meta-analysis. Eur. Urol..

[57] Yin L., Hu Q. (2014). CYP17 inhibitors—Abiraterone, C17,20-lyase inhibitors and multi-targeting agents. Nat. Rev. Urol..

[58] Cao B., Kim M., Reizine N.M., Moreira D.M. (2023). Adverse Events and Androgen Receptor Signaling Inhibitors in the Treatment of Prostate Cancer: A Systematic Review and Multivariate Network Meta-analysis. Eur. Urol. Oncol..

[59] Beer T.M., Armstrong A.J., Rathkopf D.E., Loriot Y., Sternberg C.N., Higano C.S., Iversen P., Bhattacharya S., Carles J., Chowdhury S. (2014). Enzalutamide in Metastatic Prostate Cancer before Chemotherapy. N. Engl. J. Med..

[60] Nadal R., Taplin M.-E., Bellmunt J. (2014). Enzalutamide for the Treatment of Prostate Cancer: Results and Implications of the AFFIRM Trial. Futur. Oncol..

[61] Omer D.M., Shah F., Luthra A., Chen C.-T., Lee C.I., Williams H., Walch H., Verheij F.S., Rosen R., Alvarez J. (2025). Clinical and Genomic Characterization of Secondary Rectal Cancer After Radiotherapy for Prostate Cancer. JAMA Netw. Open.

[62] Monda S., Pratsinis M., Lui H., Noel O., Chandrasekar T., Evans C.P., Dall’Era M.A. (2024). Secondary Bladder Cancer After Prostate Cancer Treatment: An Age-matched Comparison Between Radiation and Surgery. Eur. Urol. Focus.

[63] Demus T., Getzenberg R.H., Nieder A.M. (2023). Understanding Prescribing Differences Between Urologists and Medical Oncologists in the Management of Advanced Prostate Cancer. Urol. Pr..

[64] Caram M.E., Kaufman S.R., Modi P.K., Herrel L., Oerline M., Ross R., Skolarus T.A., Hollenbeck B.K., Shahinian V. (2019). Adoption of Abiraterone and Enzalutamide by Urologists. Urology.

[65] Heath E.I., Dyson G.E., Cackowski F.C., Hafron J., Powell I. (2022). Treatment Intensification Patterns and Utilization in Patients with Metastatic Castration-Sensitive Prostate Cancer. Clin. Genitourin. Cancer.

[66] Liao Y., Grobholz R., Abel U., Trojan L., Michel M.S., Angel P., Mayer D. (2003). Increase of AKT/PKB expression correlates with gleason pattern in human prostate cancer. Int. J. Cancer.

[67] McMenamin M.E., Soung P., Perera S., Kaplan I., Loda M., Sellers W.R. (1999). Loss of PTEN expression in paraffin-embedded primary prostate cancer correlates with high Gleason score and advanced stage. Cancer Res..

[68] Bitting R.L., Armstrong A.J. (2013). Targeting the PI3K/Akt/mTOR pathway in castration-resistant prostate cancer. Endocr. Relat. Cancer.

[69] Fizazi K., George D.J., De Santis M., Clarke N., Fay A.P., Uemura H., Grinsted L., Rooney C., Verheijen R., Anjum R. (2021). A phase III trial of capivasertib and abiraterone versus placebo and abiraterone in patients with de novo metastatic hormone-sensitive prostate cancer characterized by PTEN deficiency (CAPItello-281). J. Clin. Oncol..

[70] Chi K.N., Agarwal N., Armstrong A.J., Hellmis E., Schlürmann F., Sugimoto M., Ürün Y., Xing N., Aregay M., Lima J. (2024). Phase III, double-blind, placebo-controlled, 2-cohort, randomized study of saruparib (AZD5305) in combination with new hormonal agents in patients with metastatic castration-sensitive prostate cancer with and without homologous recombination repair mutation (EvoPAR-Prostate01). J. Clin. Oncol..

[71] Rathkopf D.E., Chi K.N., Olmos D., Cheng H.H., Agarwal N., Graff J.N., Sandhu S.K., Hayreh V., Lopez-Gitlitz A., Francis P.S.J. (2021). AMPLITUDE: A study of niraparib in combination with abiraterone acetate plus prednisone (AAP) versus AAP for the treatment of patients with deleterious germline or somatic homologous recombination repair (HRR) gene-altered metastatic castration-sensitive prostate cancer (mCSPC). J. Clin. Oncol..

[72] Gu W., Han W., Luo H., Zhou F., He D., Ma L., Guo H., Liang C., Chong T., Jiang J. (2022). Rezvilutamide versus bicalutamide in combination with androgen-deprivation therapy in patients with high-volume, metastatic, hormone-sensitive prostate cancer (CHART): A randomised, open-label, phase 3 trial. Lancet Oncol..

[73] Comparing a 6-month vs. Long-term Course of Rezvilutamide With ADT Plus Chemotherapy in mHSPC. https://clinicaltrials.gov/study/NCT05956639?term=Comparing%20a%206-month%20vs%20Long-term%20Course%20of%20Rezvilutamide%20With%20ADT%20Plus%20Chemotherapy%20in%20mHSPC.&rank=1.

[74] Abello A., Kenney P.A., Libertino J.A., Gee J.R. (2020). Unified Approaches to Surgery and Systemic Therapy for Renal Cell Carcinoma. Renal Cancer: Contemporary Management.

[75] Fan J., Xu K., Jiang Z., Gan C., Song H., Gao G., Wang G., Kang Q., Luo L., Wang Z. (2025). Role of 18F-PSMA-1007 PET/CT-derived quantitative volumetric tumor parameters in cytoreductive radical prostatectomy selection for patients with low-volume metastatic hormone-sensitive prostate cancer: A retrospective study. BMC Cancer.

[76] Heidenreich A., Pfister D., Porres D. (2015). Cytoreductive Radical Prostatectomy in Patients with Prostate Cancer and Low Volume Skeletal Metastases: Results of a Feasibility and Case-Control Study. J. Urol..

[77] Culp S.H., Schellhammer P.F., Williams M.B. (2014). Might Men Diagnosed with Metastatic Prostate Cancer Benefit from Definitive Treatment of the Primary Tumor? A SEER-Based Study. Eur. Urol..

[78] Antwi S., Everson T.M. (2014). Prognostic impact of definitive local therapy of the primary tumor in men with metastatic prostate cancer at diagnosis: A population-based, propensity score analysis. Cancer Epidemiol..

[79] Cytoreductive Prostatectomy Combined With Triple or Dual Systemic Therapy in mHSPC Patients. https://clinicaltrials.gov/study/NCT06350825?term=Cytoreductive%20Prostatectomy%20Combined%20With%20Triple%20or%20Dual%20Systemic%20Therapy%20in%20mHSPC%20Patients.&rank=1.

[80] Fang A.M., MacDonald L.P., Gregg J.R., Siddiqui B.A., Tang C., Chapin B.F. (2025). Prostatectomy and other local treatments for oligometastatic prostate cancer: Recent and ongoing trials. Curr. Opin. Urol..

[81] A Trial of Immunotherapy Strategies in Metastatic Hormone-sensitive Prostate Cancer. https://clinicaltrials.gov/study/NCT03879122?term=A%20Trial%20of%20Immunotherapy%20Strategies%20in%20Metastatic%20Hormone-sensitive%20Prostate%20Cancer.&rank=1.

[82] Trujillo B., Wu A., Wetterskog D., Attard G. (2022). Blood-based liquid biopsies for prostate cancer: Clinical opportunities and challenges. Br. J. Cancer.

[83] Crocetto F., Russo G., Di Zazzo E., Pisapia P., Mirto B.F., Palmieri A., Pepe F., Bellevicine C., Russo A., La Civita E. (2022). Liquid Biopsy in Prostate Cancer Management—Current Challenges and Future Perspectives. Cancers.

[84] ProBio: A Biomarker Driven Study in Patients With Metastatic Prostate Cancer (ProBio). https://clinicaltrials.gov/study/NCT03903835?term=ProBio&rank=1.

